# Biventricular systolic and diastolic function in a cohort of juvenile-onset systemic lupus erythematosus patients

**DOI:** 10.1186/1546-0096-9-S1-P260

**Published:** 2011-09-14

**Authors:** Mariana Rodrigues, Luzia Sampaio, Cláudia Moura, Patrícia Costa, Iva Brito

**Affiliations:** 1 Pediatric Rheumatology Unit, Hospital São João, Porto, Portugal; 2 Pediatric Cardiology Department; Hospital São João, Porto, Portugal; 3 Oporto Faculty of Medicine, Portugal

## Background

Cardiovascular events are the most common single cause of death in systemic lupus erythematosus (SLE). Several studies have described impaired systolic and diastolic function in adults with SLE.

## Aim

The aim of our study was to study left (LV) and right (RV) ventricular function in a population of adolescents with SLE and to discuss their clinical and prognostic implications.

## Methods

Cross-sectional and retrospective study of juvenile-SLE patientsÂ´ medical records. All patients underwent two-dimensional, M-mode, conventional Doppler and tissue Doppler imaging (TDI) to evaluate systolic and diastolic biventricular function.

## Results

13 Juvenile-SLE female patients were included, with disease onset between 6 and 16 years of age (mean 11,8 years), and mean disease duration of 6,6 years (2-15 years). One patient had moderate to severe aortic regurgitation (AoR), one had pulmonary hypertension (HTP), 5 patients had systemic hypertension (HTA); none smoked.

The majority of patients presented at least one criteria of LV diastolic dysfunction. E/A ratio was normal in 6 patients and showed restrictive pattern in 4. E/E` septal ratio showed increased left atrium pressure in 2 patients. E/Vp ratio was clearly abnormal in 3 cases, predicting high mean pulmonary capillary wedge pressure. Reduced propagation velocity of the mitral valve (Vp) was observed in 4 cases, implying impaired relaxation.

LV systolic function was preserved in all cases, with normal Tei index.

RV systolic function was normal in all patients except for one, with AoR (TDI S velocity 9,3 cm/s, tricuspid annular plane systolic excursion (TAPSE) 14,8 mm).

RV diastolic function markers were altered in some patients: decreased peak velocity of E wave in 4 patients, with increased AÂ´ wave velocity in 1 patient (HTP case). E/EÂ´and E/A ratios were normal in all cases. RV outflow tract acceleration time was decreased (implying increased mean pulmonary artery pressure) in the patient with HTP.

Due to the small sample size, no correlations with other clinical or laboratorial markers were statistically significant. BNP was increased only in the AoR patient.

## Conclusion

SLE patients can have subclinical cardiac dysfunction, and diastolic changes can be an early warning sign. Subtle abnormalities of the LV/RV diastolic function were found even in the presence of a preserved systolic function. Preclinical detection of ventricular dysfunction may identify a population at risk requiring early and aggressive interventions for the prevention of cardiovascular events.

